# Colon cancer cells secreted CXCL11 via RBP‐Jκ to facilitated tumour‐associated macrophage‐induced cancer metastasis

**DOI:** 10.1111/jcmm.16989

**Published:** 2021-10-16

**Authors:** Mengjie Liu, Xiao Fu, Lili Jiang, Jiequn Ma, Xiaoqiang Zheng, Shuhong Wang, Hui Guo, Tao Tian, Kejun Nan, Wenjuan Wang

**Affiliations:** ^1^ Department of Medical Oncology The First Affiliated Hospital of Xi'an Jiaotong University Xi'an, Shaanxi China; ^2^ 1^st^ Department of Medical Oncology Shaanxi Provincial Tumor Hospital Xi’an Shaanxi China

**Keywords:** colon cancer metastasis, CXCL11, RBP‐Jκ, TAMs, TGF‐β1

## Abstract

Metastasis is the main cause of colon cancer‐related deaths. RBP‐Jκ is involved in colon cancer development, but its function in colon cancer metastasis is still unclear. Tumour‐associated macrophages are the main cell components in tumour microenvironments. Here, we aimed to determine the function of RBP‐Jκ in colon cancer metastasis and its underlying mechanisms for modulating interactions between colon cancer cell and tumour‐associated macrophages. Through bioinformation analysis, we found that RBP‐Jκ was overexpressed in colon cancer tissues and associated with advanced colon cancer phenotypes, macrophage infiltration and shorter survival overall as confirmed by our patients’ data. And our patients’ data show that RBP‐Jκ expression and tumour‐associated macrophages infiltration are associated with colon cancer metastasis and are independent prognostic factors for colon cancer patients. Tumour‐associated macrophages induced colon cancer cell migration, invasion and epithelial‐mesenchymal transition through secreting TGF‐β1. Colon cancer cells with high RBP‐Jκ expression induced the expression of TGF‐β1 in tumour‐associated macrophages by secreting CXCL11. Our research revealed that colon cancer cells secreted CXCL11 via overexpression of RBP‐Jκ to enhance the expression of TGF‐β1 in tumour‐associated macrophages to further promote metastasis of colon cancer cells.

## 1 INTRODUCTION

As one of the leading causes of cancer‐related deaths, colon cancer is still a major public health issue worldwide.^1^, ^2^ Approximately 15%–25% patients were diagnosed with metastasis^3^ and 5‐year survival rate of metastasis patients is only 10%,^4^ so there is an urgent need to determine the mechanisms involved.

The recombination signal‐binding protein for immunoglobulin kappa J region (RBP‐Jκ) is a nuclear factor that is required for Notch signalling.^5^ RBP‐Jκ was originally identified from the nuclei of mouse pre‐B cells as a DNA‐binding protein, and the typical RBP‐Jκ DNA‐binding site contains immunoglobulin Jκ recombination signal sequences (5′‐CACTGTG‐3′).^6^ Notch pathway is abnormally activated in human cancer through chromosome rearrangement, activation point mutation or expression pattern change. Therefore, Notch pathway is the target of many kinds of human cancer treatment. The activation of Notch receptor can make γ‐Secretase work as hydrolyase and release the intracellular segment of Notch receptor, which can interact with RBP‐Jκ. By targeting Notch pathway with secretase inhibitor, the production of NICD is prevented and the RBP‐Jκ‐dependent transcriptional activity is regulated. Recently, RBP‐Jκ has been shown to be overexpressed in various human cancers, such as osteosarcoma,^7^ glioblastoma^8^ and squamous cell skin carcinoma,^9^ indicating that it may be an oncogene or act as a tumour promoter. But secretase inhibitor is not like knocking down RBP‐Jκ which can inhibit the proliferation of cancer cells to the greatest extent. This shows that the function of promoting tumour cell proliferation of RBP‐Jκ is not completely dependent on Notch signal.^10^ Although a recent study reported that RBP‐Jκ knockdown suppressed oral cancer cell EMT,^11^ the exact function of RBP‐Jκ in colon cancer metastasis remains unclear.

Metastasis is a complicated process that not only involves cancer cells but also the tumour microenvironment (TME).^12^ Monocytes enter tumour lesions and polarize to various subtypes of macrophage as different stimulants in the TME. Macrophage polarize into the M1 type by interferon‐gamma and lipopolysaccharide or into the M2 type (also known as TAMs) by IL‐4 and IL‐13.^13^ TAMs (tumour‐associated macrophages) play a key role in tumour development; however, the function of TAMs in colon cancer metastasis remains unclear.

Therefore, in this study, we explored the clinical significance of the expression of RBP‐Jκ and the infiltration of TAMs, and the revealed the underlining mechanisms of RBP‐Jκ and TAMs in colon cancer metastasis.

## 2 MATERIALS AND METHODS

### 2.1 Bioinformatic analysis

GEPIA (gepia2.cancer‐pku.cn/), TGCA (www.cancer.gov) and Oncomine databases (www.oncomine.org) were searched to analyse *RBP*‐*Jκ* expression in colon cancer. Expression data for *RBP*‐*Jκ* in colon cancer tissues and normal tissues were extracted and analysed. TIMER (https://cistrome.shinyapps.io/timer/) was used to analyse immune cell infiltration.

### 2.2 Patients

All protocol was approved by the Ethics Committee of Xi'an Jiaotong University. All patients provided informed consent before surgery. A total of 201 patients with colon adenocarcinoma were enrolled from The First Affiliated Hospital of Xi'an Jiaotong University between January 2011 and January 2013. Each patient was histologically diagnosed with colon adenocarcinoma. All patients did not receive adjuvant chemotherapy or radiotherapy before surgical resection. TNM stage was classified according to the eighth edition of the American Joint Committee on Cancer.^14^ Patients were regularly followed up until January 2018, and 115 patients died and 86 patients were alive till to the last follow‐up. Tissue specimens (in paraffin blocks) were retrieved from the Pathology Department and were used for immunohistochemistry (IHC).

### 2.3 Immunohistochemical staining

Formalin‐fixed and paraffin‐embedded tissue samples of human and mouse xenograft tissues were used for IHC based on a previous study.^15^ The antibodies used were shown in Table S1. The stained sections were semiquantitatively scored based on a previous study.^16^

### 2.4 Cell culture and transfection

The human colon adenocarcinoma cell lines RKO and SW480 and a human monocyte cell line, Thp‐1, were obtained from the Cell Bank of the Chinese Academy of Sciences (Shanghai, China). Tumour cells were cultured in Dulbecco's modified Eagle's medium (DMEM, Hyclon, US) containing 10% (v/v) foetal bovine serum (FBS). Thp‐1 cells were cultured in Roswell Park Memorial Institute medium‐1640 (RPMI1640, Hyclon, US) containing 10% FBS in a humidified incubator with 5% CO_2_ at 37ºC. Moreover, colon cancer cells and macrophages were cocultured in Transwell chambers with pore sizes of 0.4 or 8.0 µm (Corning, Corning, NY, USA). A lentiviral vector carrying *RBP*‐*Jκ* short hairpin RNAs (shRNA; GENECHEM Co. Ltd., Shanghai, China) was used to silence the expression of RBP‐Jκ in RKO cells, and a lentiviral vector carrying RBP‐Jκ cDNA (GENECHEM Co. Ltd.) was used to overexpress RBP‐Jκ in SW480 cells. For cell transfection, cells were seeded and grown to approximately 80% confluency and were then transfected with these lentiviruses according to the manufacturer's instructions. After 3 days, the transfected cells were further cultured in DMEM containing 4 μg/ml puromycin to establish stable cell sublines; these cells were named RKO‐shR (RKO cells with silenced RBP‐Jκ expression), RKO‐NC (negative control of RKP‐shR cells), SW480‐R (SW480 cells with RBP‐Jκ overexpression) and SW480‐NC (negative control of SW480‐R cells). Furthermore, tumour‐associated macrophages (TAMs) were plated in 6‐ or 24‐well plates for transfection with *TGF*‐*β1* small interfering RNA (GenePharma Co. Ltd., Shanghai, China) or a plasmid carrying TGF‐β1 cDNA (GenePharma) using Turbo‐Fect^TM^ (Thermo Fisher Scientific, Waltham, MA, USA). The RKO‐shR cells in 6‐well plates were also transfected with a plasmid carrying C‐X‐C motif chemokine 11 (CXCL11) cDNA (GenePharma) using Turbo‐Fect^TM^ (Thermo Fisher Scientific), and the SW480‐R cells in 6‐well plates were transfected with *CXCL11* siRNA (GenePharma) using Turbo‐Fect^TM^ (Thermo Fisher Scientific). mRNA expression was determined after 24 h by real‐time PCR (qPCR). Total cell protein was extracted for Western blot analysis after 48 h.

### 2.5 RNA isolation and qPCR

Total RNA was isolated from cells using Fast200 (Tiangen, Beijing, China) and was then reverse transcribed into cDNA using Prime Script^TM^ RT Master Mix (Takara, Shiga, Japan) according to the manufacturer's protocols. The relative expression of *RBP*‐*Jκ*, *TGF*‐*β1*, *CXCL11* and *GAPDH* mRNA was measured by qPCR with SYBR® Premix Ex Taq™ II (Perfect Real Time, Takara); the specific primer sequences are shown in Table S2. Their relative levels were quantified using the 2^(−ΔΔCT)^ method against the GAPDH level in each cell line. The experiments were performed in triplicate and were repeated three times.

### 2.6 Protein extraction and western blot analysis

Western blot analysis was performed according to a previous study.^15^ The antibodies used were shown in Table S1. The experiments were repeated three times.

### 2.7 TAMs induction and flow cytometry

To induce TAMs, Thp‐1 cells were plated in 6‐ or 24‐well plates at a density of 3 × 10^6^/ml and were treated with 100 ng/ml phorbol 12‐myristate 13‐acetate (PMA) (Sigma) in the dark for 24 h and the cells were further treated with 20 ng/ml interleukin 4 (IL‐4) (Sigma) and 20 ng/ml IL‐13 (Sigma) for 36 h.^13^, ^17^ The expression of CD68 and CD163 was used to identify type 2 TAMs. Next, TAMs were digested with an ethylene diamine tetra acetic acid‐free pancreatic enzyme solution and were collected and washed twice with phosphate‐buffered saline (PBS), incubated with FcBlock (564219, BD Biosciences, Franklin Lakes, NJ, USA) at room temperature for 10 min and incubated with CD68 monoclonal antibody (11–0689, Thermo Fisher Scientific) and CD163 monoclonal antibody (12–1639, Thermo Fisher Scientific) in the dark for 30 min at 37°C. The cells were then measured using a flow cytometer and analysed with FlowJo software (BD Biosciences).

### 2.8 Tumour cell migration and invasion assays

Tumour cell migration and invasion ability were assessed using 24‐well Transwell chambers with a pore size of 8 µm (Corning). For the invasion assay, the Transwell filters were precoated with 50 µl of 200 mg/ml Matrigel (356234, BD Biosciences). In brief, 1 × 10^5^ colon cancer cells (for the migration assay) or 3 × 10^5^ colon cancer cells (for the invasion assay) in 200 µl of FBS‐free medium were plated in the upper chamber, and the serum‐supplemented medium or TAMs were used as attractants in the lower chamber. Then, the cells were incubated at 37°C for 24 h, and cells migrating or invading the underside of the filters were fixed in 100% methanol, stained with a 0.5% crystal violet solution and counted under a microscope (Olympus Corporation, Tokyo, Japan). The means of the triplicate assays were used for statistical analysis.

### 2.9 Wound healing assay

Cells were seeded in 6‐well plates. Then, a wound was created across the entire well using a sterile pipette tip. After washing with PBS, the cells were further cultured in a serum‐free medium for an additional 48 h, and then, wound closure was measured after capturing photographic images from six randomly selected microscopic fields. The means of the triplicate assays were used for statistical analysis.

### 2.10 Enzyme‐linked immune sorbent assay

Cell culture supernatants were harvested, and the concentrations of TGF‐β1 and CXCL11 were determined using TGF‐β1 and CXCL11 ELISA kits (R&D Systems, Minneapolis, MN, USA) according to the manufacturer's protocols. The assays were conducted in triplicate and were repeated three times.

### 2.11 Animal experiments

The animal experiments in this study were approved by the Institutional Animal Care and Use Committee of Xi'an Jiaotong University. Four‐week‐old male BALB/c nude mice were purchased from the Animal Center of Xi'an Jiaotong University and housed in a specific pathogen‐free facility with free access to autoclaved food and water. The mice were randomized into 12 groups (*n* = 6) and were implanted subcutaneously in the back with the following: 1 × 10^6^ RKO‐NC, RKO‐shR, RKO, RKO mixed with macrophages (1:1), RKO‐shR mixed with M2‐NC2 cells (1:1), RKO‐shR mixed with M2‐T cells (1:1), SW480‐NC, SW480‐R, SW480, SW480 mixed with macrophages (1:1), SW480‐R mixed with M2‐NC1 (1:1), or SW480‐R mixed with M2‐siT (1:1) in 100 μl of RPMI1640/Matrigel (356234, BD Biosciences). The mice were monitored for 28 days and were then sacrificed for resection of xenografted tumours and immunohistochemical staining of the tumour sections. Additionally, another set of mice were also randomized into 12 groups (*n* = 6) for tail vein injections with 1 × 10^5^ RKO‐NC, RKO‐shR, RKO, RKO mixed with macrophages (1:1), RKO‐shR mixed with M2‐NC2 (1:1), RKO‐shR mixed with M2‐T (1:1), SW480‐NC, SW480‐R, SW480, SW480 mixed with macrophages (1:1), SW480‐R mixed with M2‐NC1 (1:1) or SW480‐R mixed with M2‐siT (1:1) in 100 μl of PBS. The mice were monitored for 8 weeks and were then sacrificed for lung resection and identification of lung metastasis after haematoxylin and eosin staining of the lung sections.

### 2.12 RNA sequencing

Total RNA prepared from RKO‐NC or RKO‐shR cells (see qPCR section) was purified and quantified for construction of RNA sequencing libraries. Then, the libraries were sequenced on the Illumina HiSeq platform; reads containing adapter or poly‐N or of low quality were removed, and high‐quality and clean reads were further analysed (i.e. they were Q20 [>96%], Q30 [>92%], with an uncertainty rate [<0.01%] for the clean data). The mapped reads were obtained using Tophat2 after alignment to the reference of the human genome. The number of mapped clean reads for each unigene was counted and then normalized to reads/kb/million reads to calculate the unigene expression levels.

### 2.13 Statistical analysis

The data are expressed as the mean ±standard deviation for each case number. Differences between groups were analysed using Student's *t* test, and differences among multiple variable groups were analysed using one way ANOVA analysis. The associations between the percentage of positive staining and clinical parameters were assessed using Pearson's χ^2^ test. Moreover, overall survival (OS), which was defined as the time from tumour diagnosis to patient death or the last follow‐up, was used to generate Kaplan‐Meier curves, was statistically analysed using the log‐rank test and was further assessed using univariate and multivariate analyses. All statistical analyses were conducted using SPSS 22.0 software (International Business Machines Corporation, Armonk, NY, USA) with two‐side analysis, and a *p* value <0.05 was considered statistically significant for levels of *p < 0.05, **p < 0.01 and ***p < 0.001.

## 3 RESULTS

### 3.1 *RBP*‐*Jκ* overexpression was associated with macrophage infiltration in colon cancer tissues

GEPIA was used to assess the expression of *RBP*‐*Jκ* in colon cancer tissues. We found that *RBP*‐*Jκ* expression in colon cancer tissues (n = 275) was significantly higher than that of normal colon tissue (*n* = 41, *p*<0.01; Figure S1A). Survival analysis showed that high expression of *RBP*‐*Jκ* indicated shorter OS (*p* = 0.048) and DFS (*p* = 0.060; Figure S1B–S1C). Then, we used Oncomine to further confirm this result (*p* < 0.001; Figure S1D–S1G). TCGA data showed that *RBP*‐*Jκ* was overexpressed in colon cancer tissues (31 pairs, *p *= 0.047; 478 tumour tissues vs 31 para‐carcinoma tissues, *p *= 0.046; Figure S1H–S1I). Next, we collected clinical parameters of 247 patients from TCGA data set. Correlation analysis showed that high expression of *RBP*‐*Jκ* was associated with depth of tumour invasion (*p* < 0.001) and distance metastasis (*p *= 0.022, Table S3). The above analysis results implicated that *RBP*‐*Jκ* was overexpressed in colon cancer tissues and was associated with metastasis.

Finally, we used TIMER to analyse the relevance between immune cell infiltration and *RBP*‐*Jκ* in colon cancer. Results showed that the macrophage infiltration was associated with overall survival of colon cancer patients. That was patients with high macrophage infiltration had a shorter overall survival than patients with low macrophage infiltration (*p *= 0.035; Figure S1J). *RBP*‐*Jκ* was positively correlated with macrophage infiltration (*r *= 0.486, *p *= 2.72e‐25; Figure S1K). Further analysis revealed that *RBP*‐*Jκ* was positively correlated with *CD163*, which is the marker of M2 type macrophage (*r *= 0.47, *p *= 1.02 × 10^−23^; Figure S1L).

### 3.2 RBP‐Jκ and CD163 were independent prognostic predictors of colon cancer patients

**FIGURE 1:**
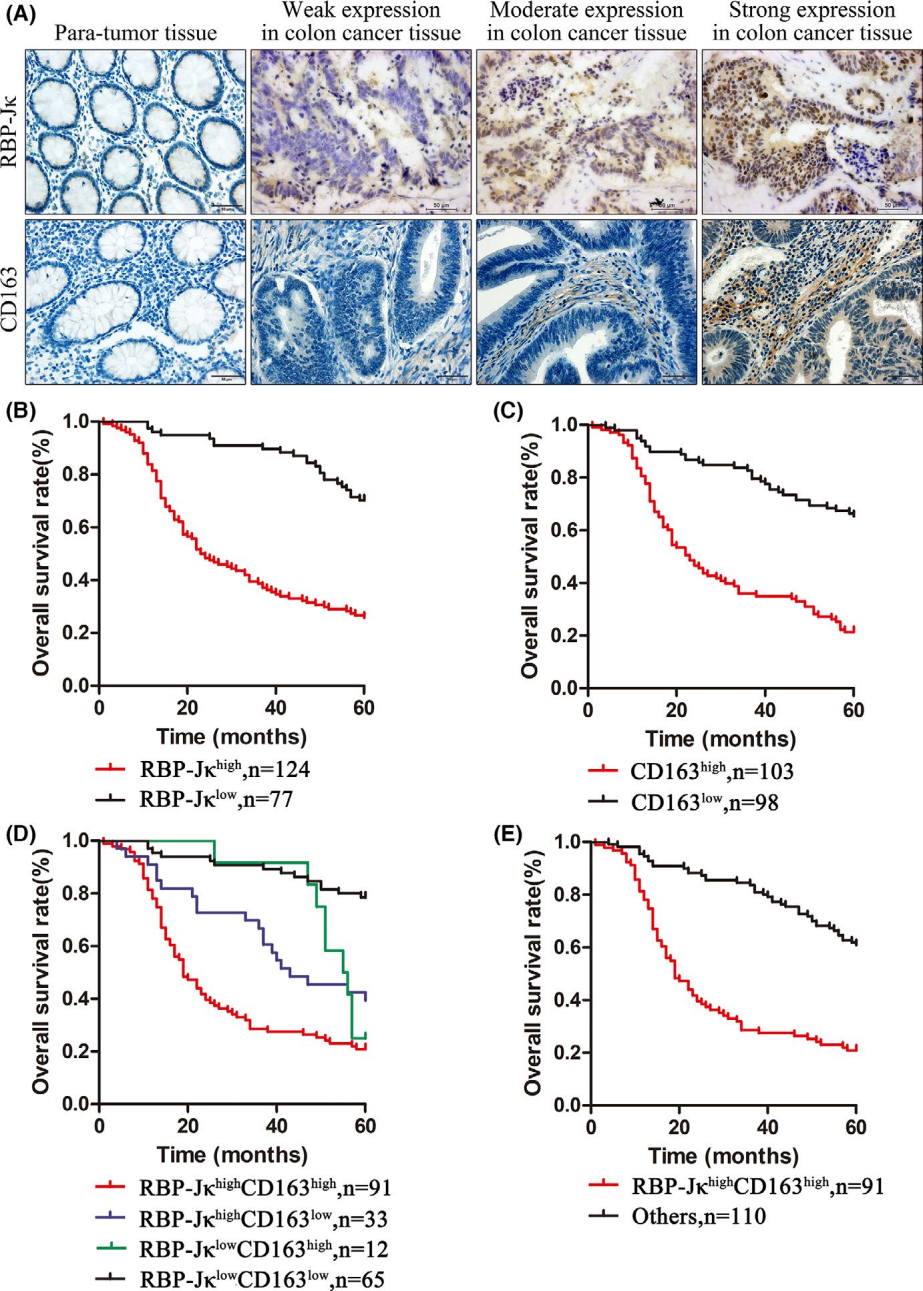
Immunohistochemical analysis of RBP‐Jκ and CD163 in colon cancer tissues. (A) Immunohistochemical staining of RBP‐Jκ and CD163. (B) Kaplan‐Meier curve survival analysis stratified by RBP‐Jκ expression. (C) Kaplan‐Meier curve survival analysis stratified by CD163 expression. (D), (E) Kaplan‐Meier curve survival analysis stratified by RBP‐Jκ and CD163 expression

To verify the results above, we compared the expression of RBP‐Jκ and CD163 in colon cancer and normal tissues from 201 patients (Figure 1A). Our IHC data showed that high RBP‐Jκ expression was observed in 124 colon cancer cases (61.69%) compared to 41 cases (20.40%) of para‐tumour tissues (*p*<0.001; Table 1). High CD163 expression was observed in 103 cases (51.24%) of colon cancer versus 42 cases (20.90%) of para‐tumour tissues, thus indicating that TAMs had more infiltration in colon cancer tissues (*p* < 0.001; Table 1). Moreover, RBP‐Jκ expression exhibited a significant positive association with CD163 (*r* = 0.562, *p* < 0.001, Table S4).

**TABLE 1:** Association of RBP‐Jκ and CD163 expression with clinicopathological features from 201 colon cancer patients

Characteristics	*n*	RBP‐Jκ			CD163		
		High	Low	*p*‐value	High	Low	*p*‐value
Histology Type							
Tumour tissue	201	124 (61.69%)	77 (38.31%)	**<0.001**	103 (51.24%)	98 (48.76%)	**<0.001**
Para‐tumour tissue	201	41 (20.40%)	160 (79.60%)		42 (20.90%)	159 (79.10%)	
Gender							
Male	125	82	43	0.14	70	55	0.084
Female	76	42	34		33	43	
Age							
<60	91	59	32	0.40	50	41	0.34
≥60	110	65	45		53	57	
Tumour location							
Right colon	113	65	48	0.16	53	60	0.16
Left colon	88	59	29		50	38	
Differentiation							
Well/Moderate	169	99	70	**0.037**	82	87	0.076
Poor	32	25	7		21	11	
Depth of invasion							
Tis/T1/T2	18	7	11	**0.037**	5	13	**0.037**
T3/T4	183	117	66		98	85	
Lymph node metastasis							
No	121	65	56	**0.004**	54	67	**0.021**
Yes	80	59	21		49	31	
Distant metastasis							
No	173	97	76	**<0.001**	82	91	**0.007**
Yes	28	27	1		21	7	
TNM Stage							
Ⅰ/Ⅱ	112	56	56	**<0.001**	47	65	**0.003**
Ⅲ/Ⅳ	89	68	21		56	33	

In addition, high RBP‐Jκ expression was associated with dedifferentiation (*p *= 0.037), invasion beyond the propria muscularis (*p* = 0.037), lymph node metastasis (*p *= 0.004), distance metastasis (*p* < 0.001) and advanced TNM stage in this cohort of patients (*p* < 0.001; Table 1). High CD163 expression was associated with invasion depth (*p *= 0.037), lymph node metastasis (*p* = 0.021), distant metastasis (*p *= 0.007) and TNM stage of patients (*p *= 0.003; Table 1). Univariate analysis results showed that high RBP‐Jκ expression (*p* < 0.001), high CD163 expression (*p* < 0.001) along with poor tumour differentiation (*p* < 0.001), invasion beyond the propria muscularis (*p* = 0.032), lymph node metastasis (*p* < 0.001), distance metastasis (*p* < 0.001) and advanced TNM stage (*p* < 0.001) were associated with shorter OS (Table 2). Multivariate analysis results revealed that RBP‐Jκ expression (*p *= 0.021), CD163 expression (*p* < 0.001), distant metastasis (*p* < 0.001) and advanced TNM stage (*p* < 0.001) were independent prognostic predictors for shorter OS (Table 2).

**TABLE 2:** Univariate and multivariate analyses of 201 colon cancer patients

Characteristics	*n*	Univariate analysis		Multivariate analysis	
		HR (95% CI)	*p*‐value	HR (95% CI)	*p*‐value
Gender					
Male	125	1	0.21		
Female	76	0.782(0.531–1.151)			
Age					
<60	91	1	0.46		
≥60	110	0.871(0.604–1.256)			
Tumour location					
Right colon	113	1	0.82		
Left colon	88	1.043(0.721–1.508)			
Differentiation					
Well/Moderate	169	1	**<0.001**		
Poor	32	2.198(1.424–3.392)			
Depth of invasion					
Tis/T1/T2	18	1	**0.032**		
T3/T4	183	2.667(1.088–6.538)			
Lymph node metastasis					
No	121	1	**<0.001**		
Yes	80	3.174(2.182–4.617)			
Distant metastasis					
No	173	1	**<0.001**	1	**<0.001**
Yes	28	6.047(3.783–9.665)		2.540(1.522–4.238)	
TNM Stage					
Ⅰ/Ⅱ	112	1	**<0.001**	1	**<0.001**
Ⅲ/Ⅳ	89	4.105(2.776–6.070)		2.964(1.924–4.566)	
RBP‐Jκ					
High	124	1	**<0.001**	1	**0.021**
Low	77	0.233(0.147–0.370)		0.542(0.322–0.912)	
CD163					
High	103	1	**<0.001**	1	**<0.001**
Low	98	0.270(0.180–0.406)		0.421(0.269–0.657)	

Moreover, patients with high tumour RBP‐Jκ expression had significantly shorter OS (*p* < 0.001; Figure 1B) and patients with high CD163 expression in colon cancer had a significantly shorter OS (*p* < 0.001; Figure 1C). Furthermore, combining RBP‐Jκ expression and CD163 expression, stratification analysis showed that patients with high RBP‐Jκ expression and high CD163 expression had the shortest OS (*p* < 0.001, Figure 1D, 1E).

### 3.3 RBP‐Jκ promoted colon cancer cell metastasis

**FIGURE 2:**
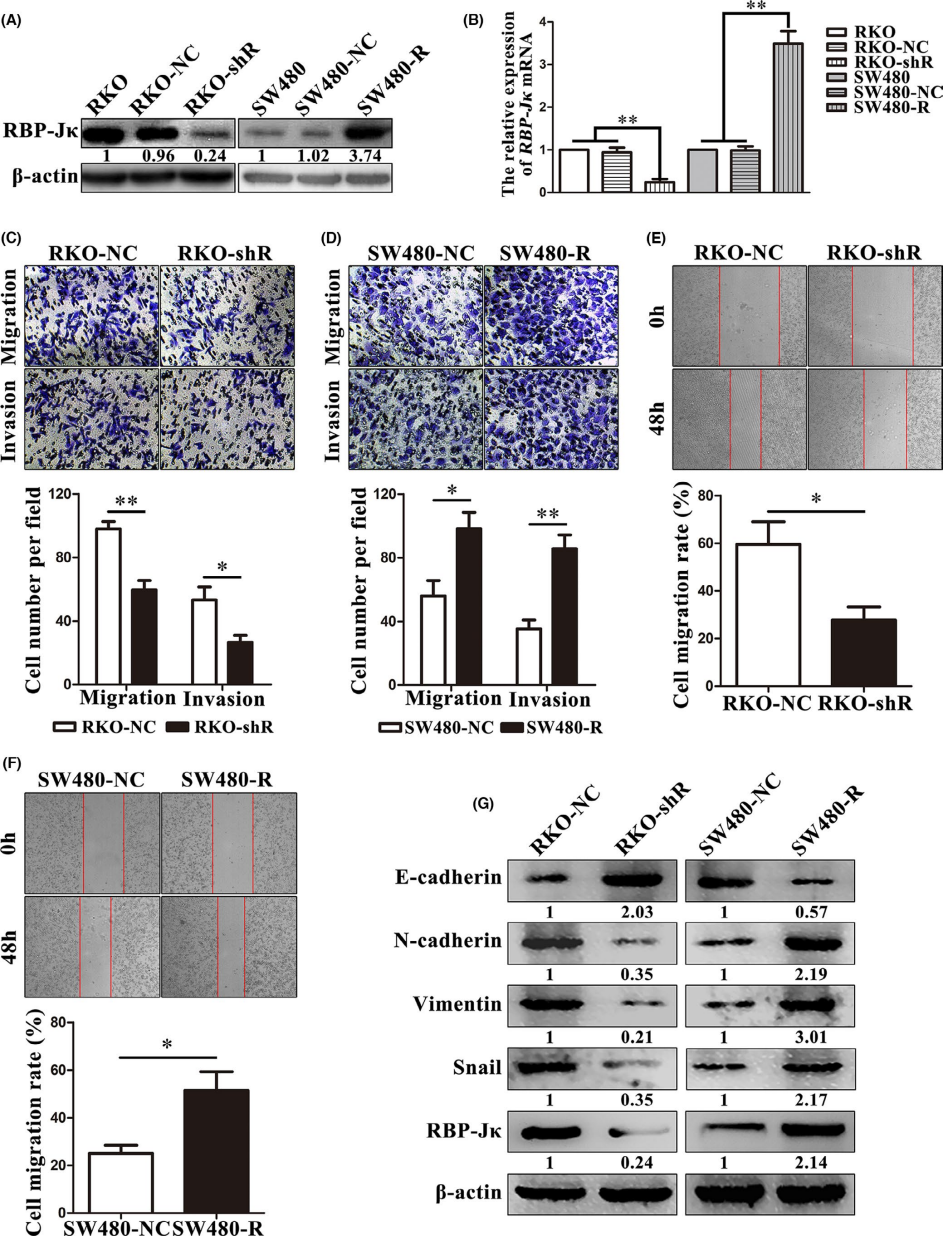
RBP‐Jκ promotes colon cancer cell metastasis *in vitro*. (A) Western blot analysis. RBP‐Jκ protein expression was lower in RKO‐shR than that in RKO and RKO‐NC cells, whereas RBP‐Jκ protein expression was higher in SW480‐R than that in SW480 and SW480‐NC cells. (B) Real‐time PCR analysis. RBP‐Jκ mRNA expression was lower in RKO‐shR than that in RKO and RKO‐NC cells, whereas RBP‐Jκ mRNA expression was higher in SW480‐R than that in SW480 and SW480‐NC cells. (C)–(D) Transwell migration and invasion assays. Silencing of RBP‐Jκ expression inhibited RKO cell migration and invasion. Overexpression of RBP‐Jκ promoted SW480 cell migration and invasion. (E)–(F) Wound healing assay. Silencing of RBP‐Jκ expression reduced the migration rate of RKO cells. Overexpression of RBP‐Jκ increased the migration rate of SW480 cells. (G) Western blot analysis. Silencing of RBP‐Jκ expression inhibited RKO cell EMT, whereas overexpression of RBP‐Jκ promoted SW480 cell EMT

Our ex vivo data revealed that RBP‐Jκ expression was associated with colon cancer metastasis. Therefore, we established stable sublines of RKO‐shR, RKO‐NC, SW480‐R and SW480‐NC cells (Figure 2A, 2B, Figure S4A). Then, we found that RBP‐Jκ knockdown significantly reduced tumour cell migration (59.67±8.18 cells per microscopic field in RKO‐shR vs. 98.00 ± 6.53 in RKO‐NC, *p *= 0.007; Figure 2C) and that RBP‐Jκ overexpression markedly induced tumour cell migration in SW480‐R vs. SW480‐NC (98.33 ± 17.56 vs. 56.00 ± 16.82, *p *= 0.039; Figure 2D). Moreover, tumour cell invasion ability was similar for RKO‐shR vs. RKO‐NC (26.67 ± 6.24 vs. 53.33 ± 11.47, *p *= 0.045; Figure 2C) and for SW480‐R vs. SW480‐NC (85.67±15.04 vs. 35.33±9.61, *p *= 0.008; Figure 2D). In addition, the wound healing assay showed that RKO‐shR had a significantly lower cell migration rate than RKO‐NC (27.74% ±7.85% vs. 59.49% ±13.44%, *p *= 0.045; Figure 2E), whereas RBP‐Jκ overexpression dramatically induced tumour cell migration for SW480‐R vs. SW480‐NC (51.54% ±13.50% vs. 25.05% ±6.00%, *p *= 0.036; Figure 2F). Because EMT contributes to increased tumour cell migration and invasion, we assessed the expression of EMT‐related proteins (e.g. E‐cadherin, N‐cadherin, Vimentin and Snail) in these cell sublines. E‐cadherin expression was downregulated but N‐cadherin, Vimentin and Snail expression was upregulated in RKO‐shR compared to RKO‐NC cells, whereas the expression pattern was reverse in SW480‐R vs. SW480‐NC cells (Figure 2G, Figure S4B).

**FIGURE 3:**
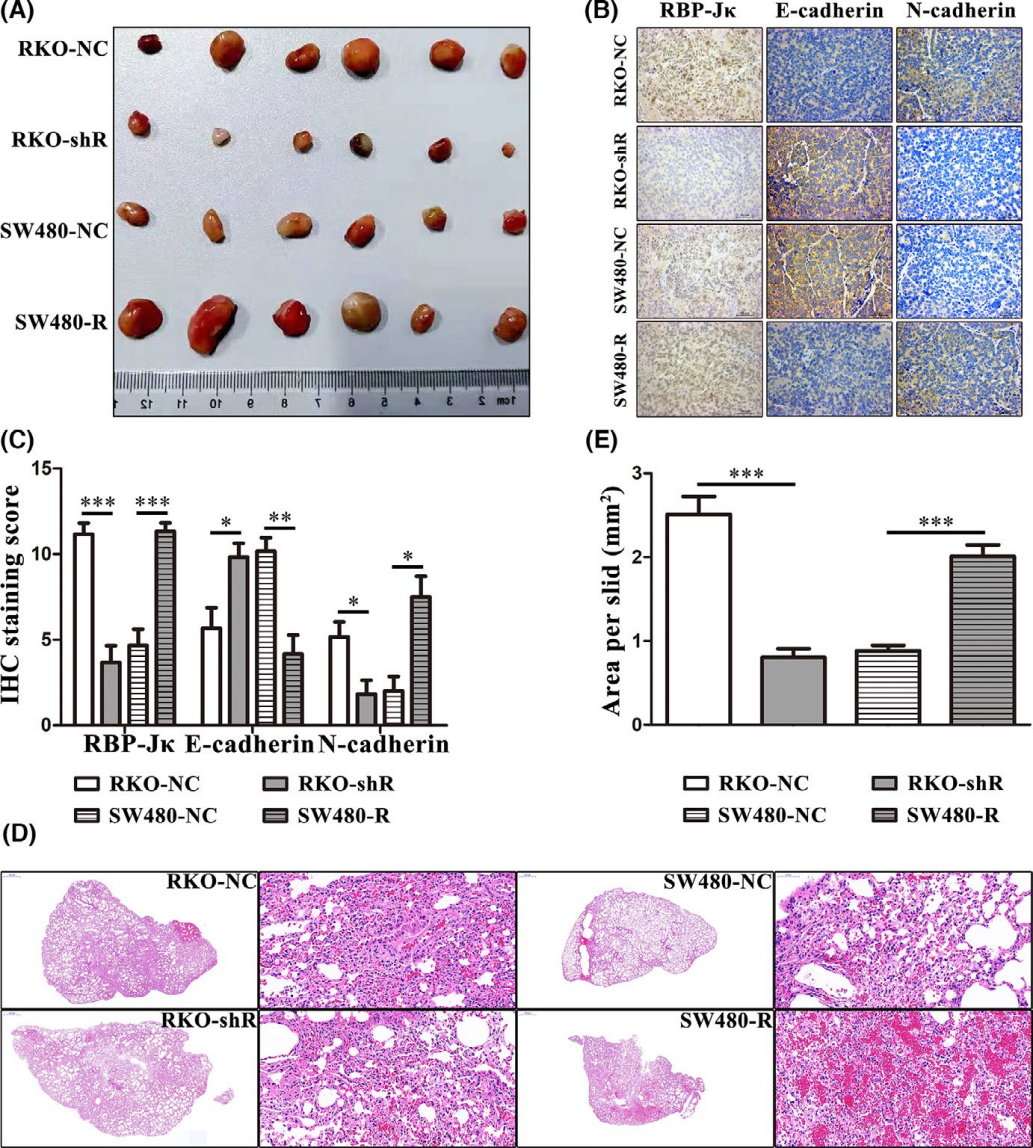
RBP‐Jκ promotes colon cancer cell metastasis *in vivo*. (A) A gross specimen of nude mouse xenograft tumours. (B)–(C) IHC. Silencing of RBP‐Jκ expression in colon cancer cells resulted in upregulation of E‐cadherin and downregulation of N‐cadherin expression in tumour cell xenografts, whereas overexpression of RBP‐Jκ downregulated the level of E‐cadherin but upregulated the level of N‐cadherin in tumour cell xenografts. (D)–(E) Nude mouse lung metastasis model. Silencing of RBP‐Jκ expression inhibited RKO cells from forming lung metastases, whereas overexpression of RBP‐Jκ promoted SW480 cells to form lung metastases. *p < 0.05, **p < 0.01 and ***p < 0.001; NC, negative control

We next evaluated the effect of RBP‐Jκ on cell EMT *in vivo*. First, we established a xenograft tumour model (Figure 3A) in nude mice using RKO‐shR, RKO‐NC, SW480‐R and SW480‐NC cells. Then, we assessed the expression of E‐cadherin and N‐cadherin in xenograft tumour tissue sections and found that the expression of E‐cadherin was lower in RKO‐NC and SW480‐R than that in RKO‐shR and SW480‐NC, while the expression of N‐cadherin was higher in RKO‐NC and SW480‐R than that in RKO‐shR and SW480‐NC (Figure 3B, 3C). Moreover, we established lung metastasis model (Figure 3D) in nude mice using RKO‐shR, RKO‐NC, SW480‐R and SW480‐NC cells to evaluated the effect of RBP‐Jκ on metastasis *in vivo*. We found that RKO‐shR cells formed smaller and fewer metastasis nodules in the lungs than RKO‐NC cells, whereas SW480‐R cells formed larger and more metastasis nodules than SW480‐NC cells (Figure 3E). Taken together, these data indicate that RBP‐Jκ expression promotes colon cancer cell metastasis both *in vitro* and *in vivo*.

### 3.4 TAMs promoted colon cancer cell metastasis by secreting TGF‐β1

As high CD163 expression was associated with cancer metastasis, we assessed the effects of CD68^+^CD163^+^TAMs on colon cancer cells. We induced Thp‐1 into TAMs and the CD68 and CD163 double‐positive rate of TAMs was 87.85% ±7.19% (Figure S2A). Next, we conducted cell migration, invasion and wound healing assays in RKO and SW480 cells cocultured with TAMs (Figure S2B). The numbers of cells that migrated (65.67 ± 7.51 vs. 89.67 ± 10.02 in RKO; *p *= 0.029; 43.33 ± 9.61 vs. 82.33 ± 12.01 in SW480; *p *= 0.012) and invaded (58.00 ± 8.00 vs. 98.33 ± 11.06 in RKO; *p *= 0.007; 46.67 ± 9.07 vs. 85.33 ± 14.01 in SW480; *p *= 0.016) increased after coculturing with TAMs (Figure S2C). The wound healing assay showed similar data (53.31% ±8.17% vs. 79.46% ±7.19% for RKO; *p *= 0.014; 21.39% ±6.06% vs. 45.36% ±8.96% for SW480; *p *= 0.018) after coculturing with TAMs (Figure S2D). Meanwhile, Western blot analysis results showed that TAMs were able to induce RKO and SW480 cells to undergo EMT (Figures S2E, S4M).

**FIGURE 4:**
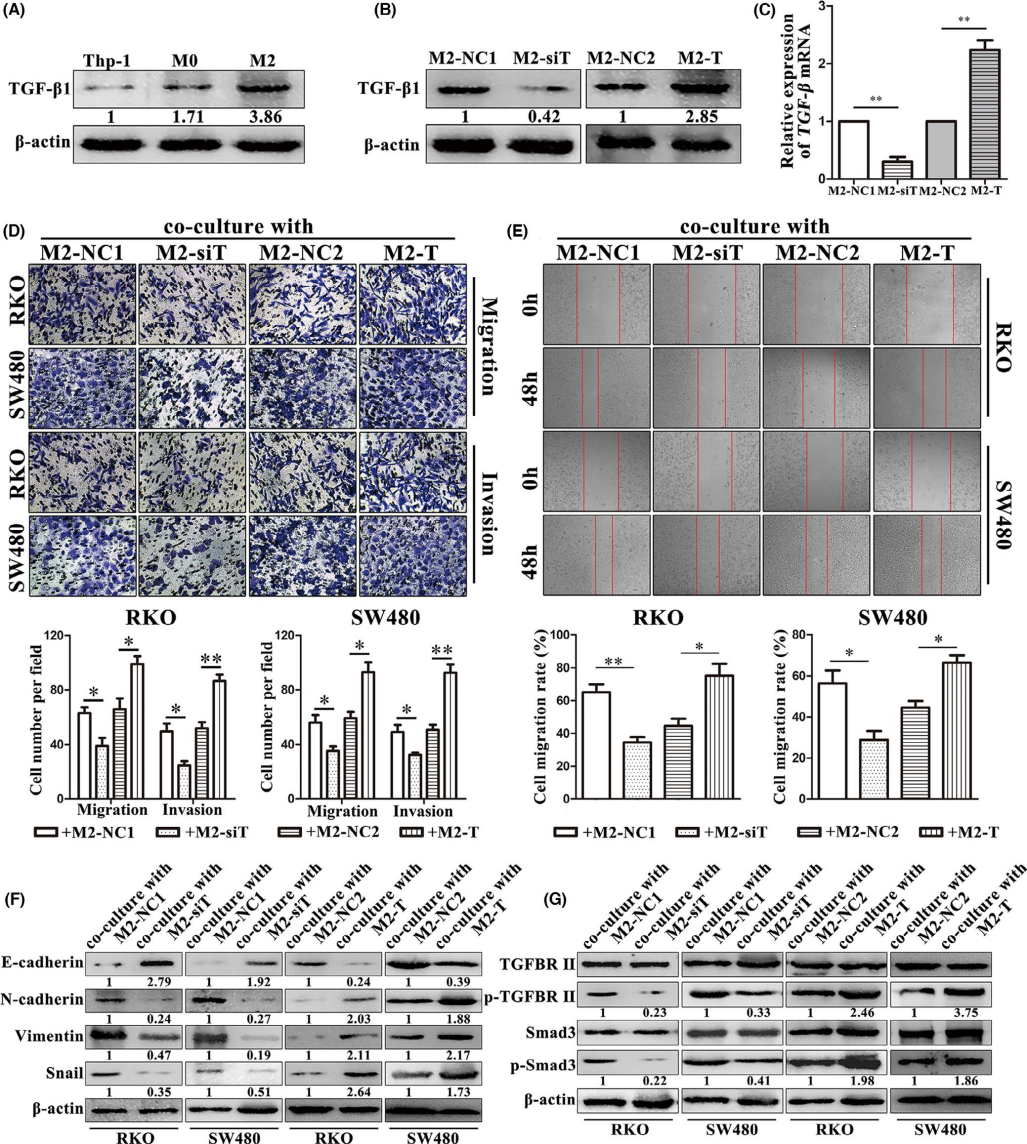
TAMs promote colon cancer cell metastasis through TGF‐β1 *in vitro*. (A) Western blot analysis. M2 type TAMs expressed more TGF‐β1 vs. M1 or monocytes. (B) Western blot analysis. TGF‐β1 was silenced and overexpressed in M2 type TAMs after transfection of TGF‐β1 siRNA and cDNA, respectively. (C) Real‐time PCR analysis. TGF‐β1 mRNA was silenced and overexpressed in M2 type TAMs after transfection of TGF‐β1 siRNA and cDNA, respectively. (D) Transwell migration and invasion assays. Silencing or overexpression of TGF‐β1 in TAMs impaired or enhanced the ability of RKO and SW480 cell migration and invasion, respectively. (E) Wound healing assay. Silencing or overexpression of TGF‐β1 in TAMs impaired or enhanced the ability of RKO and SW480 cells to migrate, respectively. (F) Western blot analysis. Silencing and overexpression of TGF‐β1 in TAMs impaired and enhanced EMT of RKO cells and SW480 cell, respectively. (G) Western blot analysis. Silencing or overexpression of TGF‐β1 in TAMs suppressed or activated the TGF‐β/Smad3 pathway in RKO and SW480 cells, respectively

Furthermore, we found that TGF‐β1 expression gradually increased during the TAMs induction process (Figure 4A, Figure S4C). Thus, we established TGF‐β1‐knockdown TAMs (M2‐siT) or negative control cells (M2‐NC1) and TGF‐β1‐overexpressed TAMs (M2‐T) or negative control cells (M2‐NC2; Figure 4B, 4C, Figure S4D). We then cocultured RKO and SW480 cells with M2‐siT or M2‐T for use in cell migration, invasion and wound healing assays. The numbers of cells that migrated (63.00 ± 7.55 vs. 39.00 ± 10.15 for RKO; *p* = 0.030; 56.00 ± 9.54 vs. 35.33 ± 5.69 for SW480; *p* = 0.032; Figure 4D) and invaded (49.67 ± 10.02 vs. 24.67 ± 5.51 for RKO; *p* = 0.019; 49.00 ± 9.17 vs. 32.33 ± 3.06 for SW480; *p* = 0.040; Figure 4D) and the cell migration rate (65.02% ±8.31% vs. 34.50% ±5.60% for RKO; *p* = 0.006; 56.32% ±10.94% vs. 28.84% ±7.48% for SW480; *p* = 0.023; Figure 4E) all decreased after coculturing with M2‐siT. In contrast, the numbers of tumour cells that migrated (66.00 ± 13.53 vs. 99.00 ± 10.15 for RKO; *p* = 0.028; 59.33 ± 8.08 vs. 93.00 ± 12.53 for SW480; *p* = 0.017; Figure 4D) and invaded (52.00 ± 7.55 vs. 86.67 ± 8.02 for RKO; *p* = 0.006; 50.67 ± 6.51 vs. 92.67 ± 10.50 for SW480; *p* = 0.004; Figure 4D) and the cell migration rate (44.61% ±7.59% vs. 75.19% ±12.48% for RKO; *p* = 0.022; 44.55% ±5.65% vs. 66.37% ±6.19% for SW480; *p* = 0.011; Figure 4E) were induced after coculturing with M2‐T. Moreover, our Western blot analysis results also showed that knockdown of TGF‐β1 expression in TAMs reversed the effect of TAMs on tumour cell EMT and that TGF‐β1 overexpression in TAMs facilitated this effect (Figure 4F, Figure S4E). In addition, TAMs activated the TGF‐β/Smad3 pathway in both RKO and SW480 cells (Figure 4G, Figure S4F).

**FIGURE 5:**
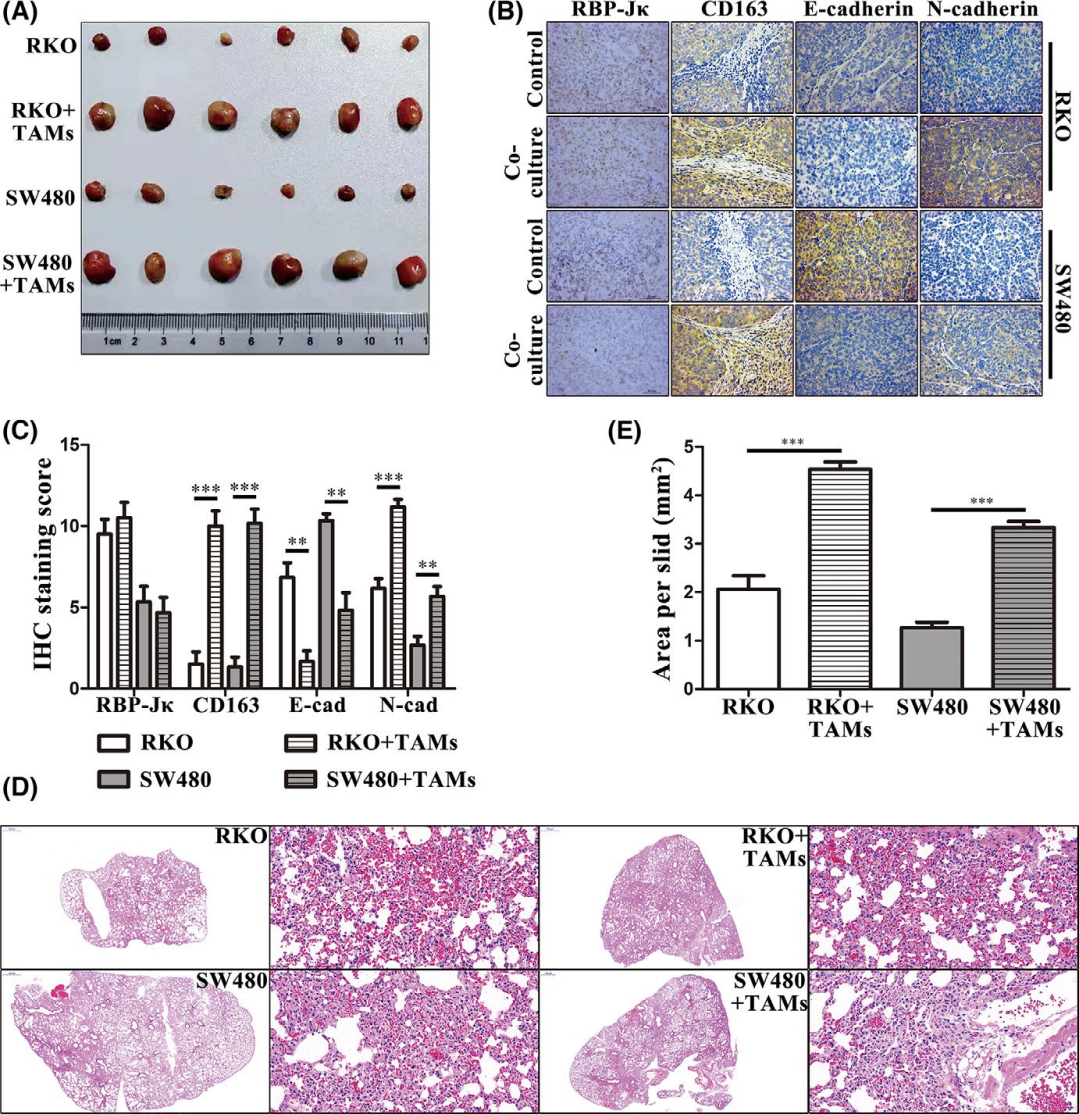
TAMs promote colon cancer cell metastasis through TGF‐β1 *in vivo*. (A) A gross specimen of nude mouse xenograft tumours. (B‐C) IHC. Expression of CD163, E‐cadherin and N‐cadherin in xenograft tumours was altered by M2 type TAMs (i.e. E‐cadherin expression was reduced, whereas the levels of N‐cadherin were induced in xenografts). (D‐E) Nude mouse lung metastasis model. TAMs promoted formation of lung metastases. *p < 0.05, **p < 0.01 and ***p < 0.001; NC, negative control

In addition, we also assessed the effect of TAMs on the induction of colon cancer cell EMT in the nude mouse xenograft tumour (Figure 5A) by using RKO or SW480 cells with or without macrophages. CD163 immunostaining of the mesenchyme in the cocultured group showed higher expression than that of the RKO or SW480‐alone groups, whereas E‐cadherin immunostaining in the cocultured group was lower than the RKO or SW480‐alone groups. The expression of N‐cadherin was higher in the cocultured group than that in the RKO or SW480‐alone groups (Figure 5B, 5C). Lung metastasis models (Figure 5D) were established by using RKO or SW480 cells with or without macrophages. Similarly, the lung metastasis model provided comparable data, that is RKO cells or SW480 cells formed smaller and fewer metastasis nodules in the lungs than that with macrophages (Figure 5E).

### 3.5 RBP‐Jκ promoted colon cancer cell metastasis through inducing TAMs secret TGF‐β1

**FIGURE 6:**
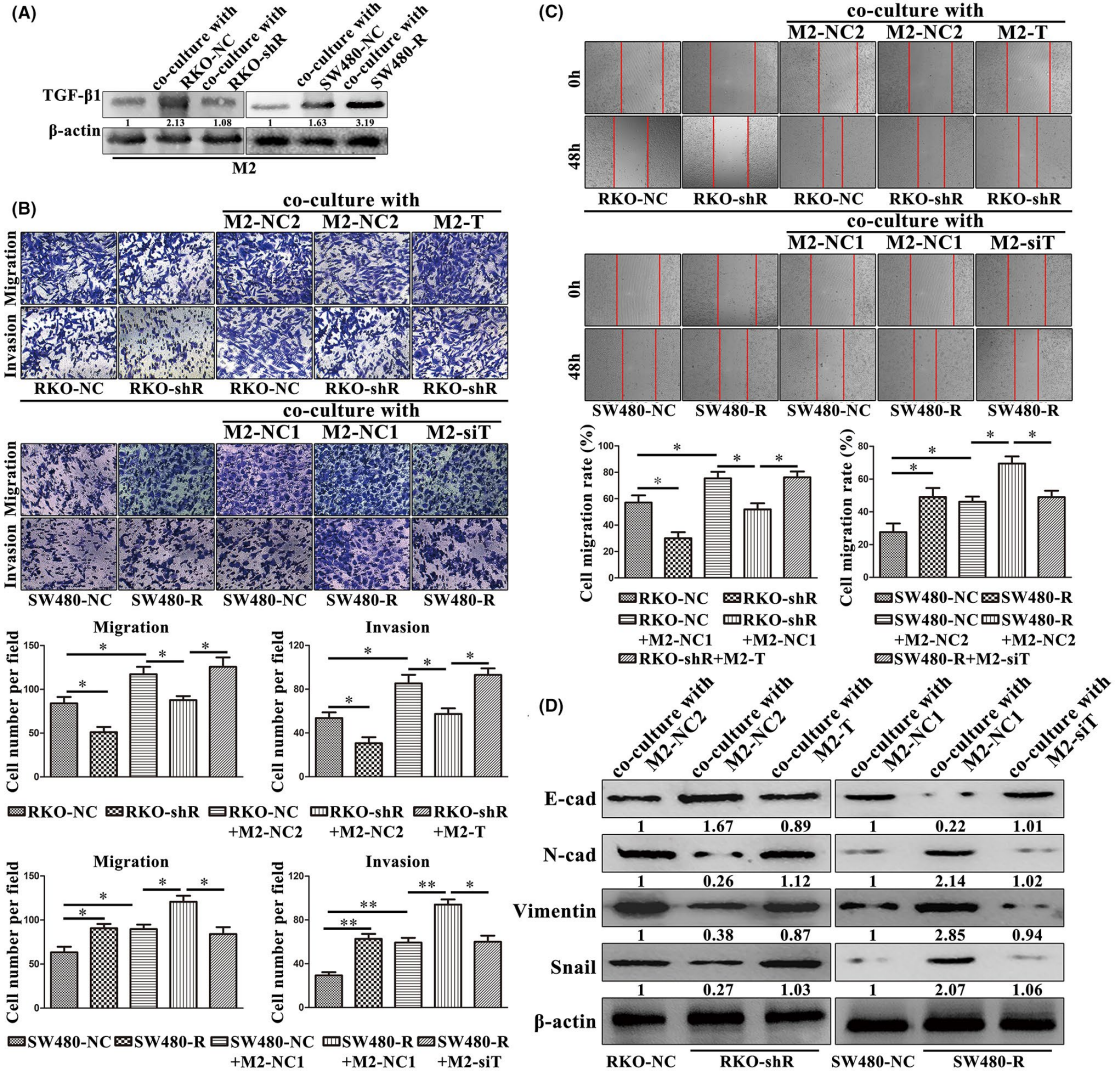
RBP‐Jκ promotes colon cancer cell metastasis through the induction of TGF‐β1 expression in TAMs *in vitro*. (A) Western blot analysis. Silencing or overexpression of RBP‐Jκ suppressed or enhanced M2 type TAMs expression of TGF‐β1, respectively. (B) Transwell migration and invasion assays. TGF‐β1 overexpression in TAMs reversed the effect of RBP‐Jκ silencing and resulted in the promotion of RKO‐shR cell migration and invasion. Silencing of TGF‐β1 in TAMs reversed the effect of RBP‐Jκ overexpression and resulted in the inhibition of SW480‐R cell migration and invasion. (C) Wound healing assay. TGF‐β1 overexpression in TAMs reversed the effect of RBP‐Jκ silencing and resulted in the promotion of RKO‐shR cell migration. Silencing of TGF‐β1 in TAMs reversed the effect of RBP‐Jκ overexpression and resulted in the inhibition of SW480‐R cell migration. (D) Western blot analysis. TGF‐β1 overexpression in TAMs reversed the effect of RBP‐Jκ silencing and resulted in the promotion of RKO‐shR cell EMT, whereas silencing of TGF‐β1 expression in TAMs reversed the effect of RBP‐Jκ overexpression and resulted in the inhibition of SW480‐R cell EMT

Taken together, our data show that RBP‐Jκ and TAMs participate in promoting colon cancer metastasis. We then detected the relationship between colon cancer expression RBP‐Jκ and TAMs and found that TGF‐β1 expression was downregulated or upregulated in TAMs when cocultured with RKO‐shR or SW480‐R cells (Figure 6A, Figure S4G). We further evaluated whether RBP‐Jκ induced colon cancer cell migration and invasion through TGF‐β1 expression in TAMs by establishing six different coculture systems: i) RKO‐NC coculture with M2‐NC2; ii) RKO‐shR coculture with M2‐NC2; iii) RKO‐shR coculture with M2‐T; iv) SW480‐NC coculture with M2‐NC1; v) SW480‐R coculture with M2‐NC1; and vi) SW480‐R coculture with M2‐siT, for migration, invasion and wound healing assays. Our data showed that downregulation or upregulation of RBP‐Jκ expression in colon cancer cells suppressed or promoted tumour cell migration and invasion capacities, while upregulation or downregulation of TGF‐β1 expression in TAMs reversed the downregulation or upregulation effect of RBP‐Jκ on colon cancer cells (Figure 6B, 6C). Furthermore, our Western blot analysis results revealed that downregulation or upregulation of RBP‐Jκ expression in colon cancer cells inhibited or promoted tumour cells to undergo EMT, whereas upregulation or downregulation of TGF‐β1 expression in TAMs reversed the downregulation or upregulation effect of RBP‐Jκ on colon cancer cells (Figure 6D, Figure S4H).

**FIGURE 7:**
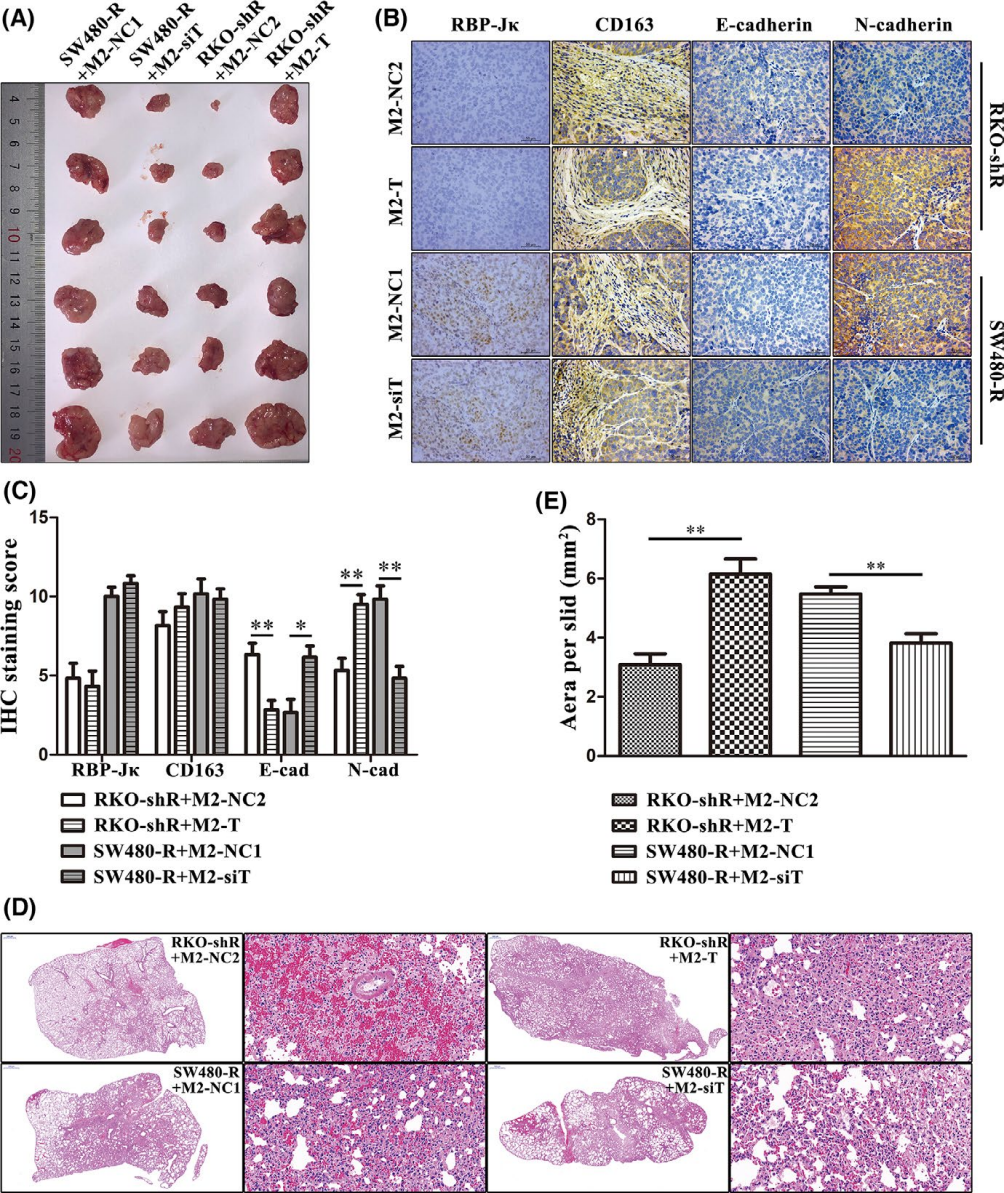
RBP‐Jκ promotes colon cancer cell metastasis through the induction of TGF‐β1 expression in TAMs *in vivo*. (A) A gross specimen of nude mouse xenograft tumours. (B‐C) IHC. TGF‐β1 overexpression in TAMs reversed the effect of silencing RBP‐Jκ and resulted in the downregulation of E‐cadherin but the upregulation of N‐cadherin, whereas silencing of TGF‐β1 expression in TAMs reversed the effect of RBP‐Jκ overexpression and resulted in the upregulation of E‐cadherin but the downregulation of N‐cadherin. (D‐E) Nude mouse lung metastasis model. TGF‐β1 overexpression in TAMs promoted the formation of lung metastases of RKO‐shR cells, whereas silencing of TGF‐β1 expression in TAMs inhibited lung metastases of SW480‐R cells. *p < 0.05 and **p < 0.01. NC, negative control

Additionally, we then established a xenograft tumour model with RKO‐shR plus M2‐NC2 or M2‐T or SW480‐R plus M2‐NC1 or M2‐siT (Figure 7A). All the tumour tissues in these four groups expressed CD163 in the mesenchyme, whereas tumours in the RKO‐shR groups expressed low levels of RBP‐Jκ and tumours in SW480‐R groups exhibited high levels of RBP‐Jκ. E‐cadherin expression in the RKO‐shR plus M2‐T group or SW480‐R plus M2‐NC1 group was lower than that in the RKO‐shR plus M2‐NC2 or SW480‐R plus M2‐siT groups, while the expression of N‐cadherin showed the opposite phenomenon (Figure 7B, 7C). Similarly, data from the lung metastasis model (Figure 7D) also confirmed these findings, which showed that metastasis nodules in the RKO‐shR plus M2‐T or SW480‐R plus M2‐NC1 groups were larger and more prevalent than those in the RKO‐shR plus M2‐NC2 or SW480‐R plus M2‐siT groups (Figure 7E). These data suggest that RBP‐Jκ expression in colon cancer cells promotes tumour cell metastasis via TAM expression of TGF‐β1.

### 3.6 Colon cancer cell with overexpressed RBP‐Jκ inducted TAMs to express TGF‐β1 by secretion of CXCL11

**FIGURE 8:**
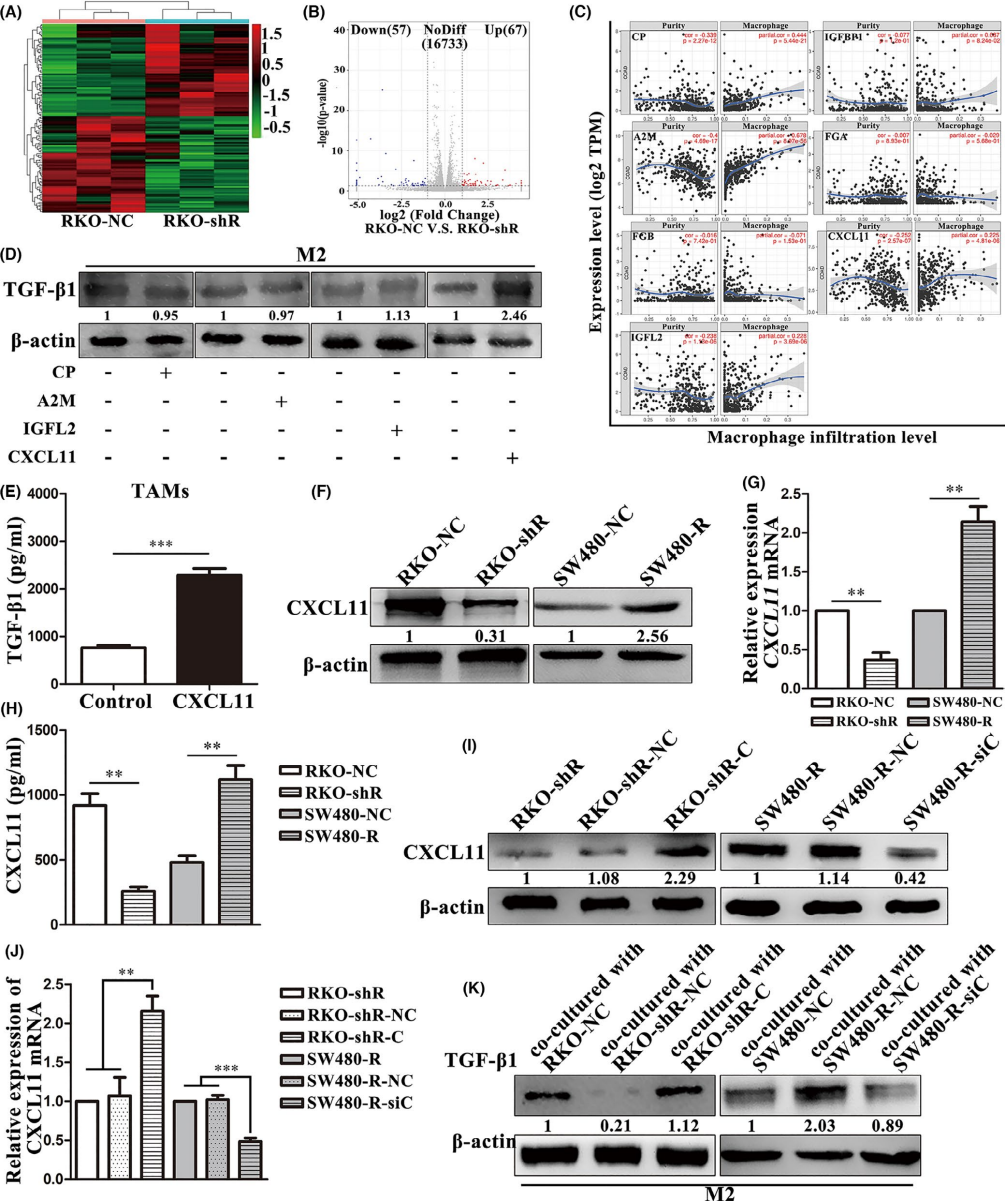
RBP‐Jκ facilitates TGF‐β1 expression of TAMs by secreting CXCL11. (A) Heat map of differentially expressed genes between RKO‐NC and RKO‐shR cells. (B) Volcano map of differentially expressed genes between RKO‐NC and RKO‐shR cells. (C) TIMER analysis. CP, A2 M, CXCL11 and IGFL2 were positively associated with macrophage infiltration. FGA and FGB were negatively associated with macrophage infiltration. IGFBP1 was unrelated to macrophage infiltration. (D) Western blot analysis. CXCL11 upregulated TGF‐β1 expression of M2 type TAMs. (E) ELISA. TGF‐β1 expression in TAMs was upregulated after CXCL11 treatment. (F) Western blot analysis. Silencing or overexpression of RBP‐Jκ downregulated or upregulated the expression of the CXCL11 protein, respectively. (G) Real‐time PCR analysis. Silencing or overexpression of RBP‐Jκ downregulated or upregulated the expression of CXCL11 mRNA, respectively. (H) ELISA. The CXCL11 concentrations in the cell culture supernatants showed that silencing or overexpression of RBP‐Jκ decreased or increased CXCL11 levels, respectively. (I) Western blot analysis. The CXCL11 protein was overexpressed or silenced in RKO‐shR and SW480‐R cells, respectively, after transfection of CXCL11 cDNA or siRNA. (J) Real‐time PCR analysis. CXCL11 mRNA was overexpressed or silenced in RKO‐shR and SW480‐R cells, respectively, after transfection of CXCL11 cDNA or siRNA. (K) Western blot analysis. TGF‐β1 expression in TAMs was up‐ or downregulated after coculturing with RKO‐shR‐C or SW480‐R‐siC cells, respectively. **p < 0.01 and ***p < 0.001. NC, negative control

We demonstrated that colon cancer cells with RBP‐Jκ overexpression induced TAMs to express TGF‐β1, but the underlying molecular events still remained to be determined. Therefore, we performed an RNA sequencing assay to identify the molecules that were possibly involved. Among the differentially expressed RNAs, there were 57 genes downregulated (Figure 8A, 8B). And among the 57 genes, there were 7 secretory proteins which were associated with immune and inflammation (CP, ceruloplasmin; A2 M, alpha‐2‐macroglobulin; CXCL11, C‐X‐C motif chemokine ligand 11; IGFL2, IGF like family member 2; FGB, fibrinogen beta chain; FGA, fibrinogen alpha chain; and IGFBP1, insulin‐like growth factor binding protein 1). Then, we used TIMER to analyse the relevance between macrophage infiltration and the 7 genes. Results showed that CP, A2 M, CXCL11 and IGFL2 were positively related to macrophage infiltration, while FGB and FGA were negatively related to macrophage infiltration, and IGFBP1 had little to do with macrophage infiltration (Figure 8C). Next, we treated TAMs separately with CP, A2 M, CXCL11 and IGFL2 and found that CXCL11 induced TAMs express TGF‐β1 (Figure 8D, Figure S4I) and the concentration of TGF‐β1 in the TAMs‐cultured supernatant after CXCL11 treatment was significantly induced (765.24 ± 82.77 vs. 2286.74 ± 241.23 pg/mL; *p*<0.001; Figure 8E). The sequencing analysis was confirmed by real‐time PCR and Western blot analyses which showed that the *CXCL11* mRNA and protein levels were lower in RKO‐shR and SW480‐NC cells than those in RKO‐NC and SW480‐R cells, respectively (Figure 8F, 8G, Figure S4J). Moreover, the RKO‐shR or SW480‐NC‐cultured supernatant concentration of CXCL11 was also significantly lower than that of RKO‐NC or SW480‐R (918.15 ± 158.34 vs. 257.94 ± 57.86 pg/mL; *p* = 0.002; 480.35 ± 87.62 vs. 1117.77 ± 188.69 pg/mL; *p* = 0.006; Figure 8H). We further demonstrated that RBP‐Jκ facilitated TAMs to express TGF‐β1 via CXCL11 secretion after overexpression of CXCL11 in RKO‐shR cells (RKO‐shR‐C) versus RKO‐shR‐NC cells as a control or after knockdown of CXCL11 expression in SW480‐R cells (SW480‐R‐siC) vs. SW480‐R‐NC cell as a control (Figure 8I, 8K, Figure S4K). We cocultured TAMs with RKO‐NC, RKO‐shR‐NC, RKO‐shR‐C, SW480‐R, SW480‐R‐NC or SW480‐R‐siC cells for Western blot analysis of TGF‐β1. We found that RKO‐shR‐C cells or SW480‐R and SW480‐R‐NC cells stimulated TAMs to express TGF‐β1 (Figure 8K, Figure S4L). These data indicate that RBP‐Jκ induces TAMs to express TGF‐β1 by increasing colon cancer cell secretion of CXCL11.

## 4 DISCUSSION

Although Notch signalling is closely associated with carcinogenesis and the development of colon cancer,^18^ studies of the effect of RBP‐Jκ, the main transcription factor of Notch pathway,^5^ on colon cancer metastasis remain scarce. Hence, in this study, we first analysed RBP‐Jκ expression in colon cancer tissues with TCGA data and found that RBP‐Jκ was overexpressed in colon cancer tissues and associated with distant metastasis, poor OS and macrophage infiltration. To strengthen our bioinformation findings, we tested RBP‐Jκ expression and TAMs infiltration in human colon cancer tissues and analysed the associations between TAMs infiltration and RBP‐Jκ expression, and the characteristics and outcomes of colon cancer patients. We consistently found that high RBP‐Jκ expression was related to more infiltration of TAMs in colon cancer tissues. Additionally, high RBP‐Jκ and more infiltration of TAMs in colon cancer tissues were associated with metastasis and poor prognosis. Our current data confirmed that RBP‐Jκ is closely associated with colon cancer progression; however, these results need to be validated using a larger cohort of patients. We then performed various *in vitro* and *in vivo* assays and determined that RBP‐Jκ overexpression induced colon cancer cell metastasis.

Different macrophage populations possess and express different molecular markers: M1 type play anti‐inflammation and antitumour roles^19^ and are characterized by the high expression of CD80^20^ and CD86,^21^ whereas M2 type play proinflammation and protumor roles^22^, ^23^, ^24^ and express high levels of CD163,^25^ CD204^26^ and CD206^27^ and play as markers of TAMs. In our study, we cocultured CD68^+^CD163^+^ TAMs with colon cancer cells with different RBP‐Jκ expression. We found that colon cancer cells with high RBP‐Jκ expression were capable of stimulating TAMs to secrete more TGF‐β1, and TAMs further facilitated EMT, migration and invasion of colon cancer cells. Hence, one or more cytokines or growth factors may mediate the signalling from RBP‐Jk in colon cancer cells to TAMs. Using an RNA sequencing assay, we found that RBP‐Jκ was able to upregulate the expression of CXCL11 in colon cancer cells. CXCL11 is a member of the CXC chemokine family.^28^ CXCL11 is the ligand with the highest affinity to CXCR3, followed by CXCL9 and CXCL10.^29^ As an ELR (Glu‐Leu‐Arg)‐negative CXC chemokine, CXCL11 can generally attenuate angiogenesis and thus have an antitumour effect.^30^ In this study, we established a xenograft tumour model for tail vein injections with RKO‐shR plus M2‐NC2(1:1) or M2‐T (1:1)or SW480‐R plus M2‐NC1(1:1) or M2‐siT (1:1), the number of cells and injectate volume were determined by our pre‐experiment according to previous studies.^31^, ^32^ However, there may be some possible limitations in this method. Although the method of establishing metastatic lung cancer model by injecting cancer cells into mouse tail vein is often used, there are many differences in the evaluation indexes of this kind of lung cancer model, and there is no systematic discussion on the window period suitable for the experiment and the repeatability of the model. Therefore, further efforts are needed to improve our metastasis model in our future research.

To our knowledge, our study being conducted at present is the first one that investigates the association between RBP‐Jκ and CXCL11 expression in colon cancer, as well as high RBP‐Jκ and CXCL11 co‐expression in relation to malignant colon cancer transformation. Interestingly, in our research, we found that in colon cancer cells, RBP‐Jκ‐induced CXCL11 was able to enhance the function of TAMs. At this point, we found a loop of colon cancer cells and TAMs in which colon cancer cells and TAMs promoted the functions of each other and thus led to colon cancer metastasis (Figure S3).

## CONFLICT OF INTEREST STATEMENT

All the authors declare that they have no competing interests.

## AUTHOR CONTRIBUTIONS

**Mengjie Liu:** Conceptualization (equal); Data curation (equal); Formal analysis (equal); Writing‐original draft (equal). **Xiao Fu:** Data curation (equal); Formal analysis (equal). **Lili Jiang:** Data curation (supporting); Formal analysis (supporting). **Jiequn Ma:** Funding acquisition (equal); Investigation (supporting). **Xiaoqiang Zheng:** Investigation (supporting). **Shuhong Wang:** Software (equal). **Hui Guo:** Supervision (equal); Writing‐review & editing (equal). **Tao Tian:** Writing‐review & editing (supporting). **Kejun Nan:** Supervision (equal). **Wenjuan Wang:** Funding acquisition (equal); Supervision (equal); Writing‐review & editing (equal).

## Supporting information

Figure S1
Figure S2
Figure S3
Figure S4
Table S1
Table S2
Table S3
Table S4

Figure 2C

## Data Availability

The data that support the findings of this study are openly available at gepia2.cancer‐pku.cn/, www.cancer.gov, www.oncomine.org, https://cistrome.shinyapps.io/timer/.
