# Supplementary material for: Colon cancer cells secreted CXCL11 via RBP‐Jκ to facilitated tumour‐associated macrophage‐induced cancer metastasis
Source: J Cell Mol Med. 2021 Oct 16;25(22):10575–90. doi: 10.1111/jcmm.16989 (PMC8581314; doi:10.1111/jcmm.16989)
Supplement: Supplementary file 1 — Figure S1 Figure S2 Figure S3 Figure S4 Table S1 Table S2 Table S3 Table S4 [file JCMM-25-10575-s001.docx]

**Supplementary Table 1. Antibodies used in this study**

| Name | Dilute Concentration | Purpose | Production ID | Manufacturer Information |
| --- | --- | --- | --- | --- |
| RBP-Jκ | 1:200 | IHC | ab25949 | Abcam, Cambridge, MA, USA |
|  | 1:1000 | WB |  |  |
| E-cadherin | 1:200 | IHC | 14472 | Cell Signaling Technology (CST), Danvers, MA, USA |
|  | 1:1000 | WB |  |  |
| N-cadherin | 1:200 | IHC | 13116 | CST |
|  | 1:1000 | WB |  |  |
| p-TGFBR II | 1:800 | WB | ab111564 | Abcam |
| p-Smad3 | 1:800 | WB | ab52903 | Abcam |
| CD163 | 1:200 | IHC | 93498 | CST |
| Snail | 1:1000 | WB | 3879 | CST |
| Vimentin | 1:1000 | WB | 5741 | CST |
| TGF-β1 | 1:1000 | WB | ab92486 | Abcam |
| TGFBRII | 1:1000 | WB | 79424 | CST |
| Smad3 | 1:1000 | WB | 9523 | CST |
| CXCL11 | 1:1500 | WB | ab9955 | Abcam |
| β-actin | 1:1000 | WB | 4970 | CST |

**Supplementary Table 2. Primer sequences**

| Gene | Forward primer | Reverse primer |
| --- | --- | --- |
| RBP-Jκ | 5'-CAAGAGTCTCAACCGTGTGCATTTA-3' | 5'-ACATGAAGTGCTTTCGCTTGTCTG-3' |
| TGF-β1 | 5'-TGAGTGGCTGTCTTTTGACG-3’ | 5'-TTCTCTGTGGAGCTGAAGCA-3' |
| CXCL11 | 5'-TGAGTGTGAAGGGCATGGCT-3' | 5'-TGAGTGTGAAGGGCATGGCT-3' |
| GAPDH | 5'-ATGATTCTACCCACGGCAAG-3' | 5'-CTGGAAGATGGTGATGGGTT-3' |

**Supplementary Table 3. Association of RBP-Jκ expression with clinicopathological features of colon cancer patients from TCGA**

| Characteristics | n | RBP-Jκ | *P*-value |
| --- | --- | --- | --- |
| Gender |  |  | **0.041** |
| Male | 127 | 7.14±2.27 |  |
| Female | 119 | 7.80±2.78 |  |
| Age (years) |  |  | 0.51 |
| <60 | 30 | 7.74±2.50 |  |
| ≥60 | 216 | 7.42±2.55 |  |
| Tumor location |  |  | 0.055 |
| Right colon | 139 | 7.73±2.65 |  |
| Left colon | 107 | 7.10±2.37 |  |
| Depth of invasion |  |  | **<0.001** |
| Tis/T1/T2 | 29 | 5.31±1.67 |  |
| T3/T4 | 216 | 7.74±2.51 |  |
| Lymph node metastasis |  |  | 0.190 |
| No | 144 | 7.28±2.67 |  |
| Yes | 102 | 7.71±2.34 |  |
| Distance metastasis |  |  | **0.022** |
| No | 219 | 7.33±2.56 |  |
| Yes | 27 | 8.51±2.13 |  |
| TNM Stage |  |  | 0.277 |
| I/II | 138 | 7.30±2.65 |  |
| III/IV | 108 | 7.66±2.40 |  |

**Supplementary Table 4. Association between RBP-Jκ and CD163 expression in 201 colon cancer patients**

|  | CD163 | | *r* | *P*-Value |
| --- | --- | --- | --- | --- |
|  | High | Low |  |  |
| RBP-Jκ |  |  |  |  |
| High | 91 | 33 | 0.562 | ＜0.001 |
| Low | 12 | 65 |  |  |

**
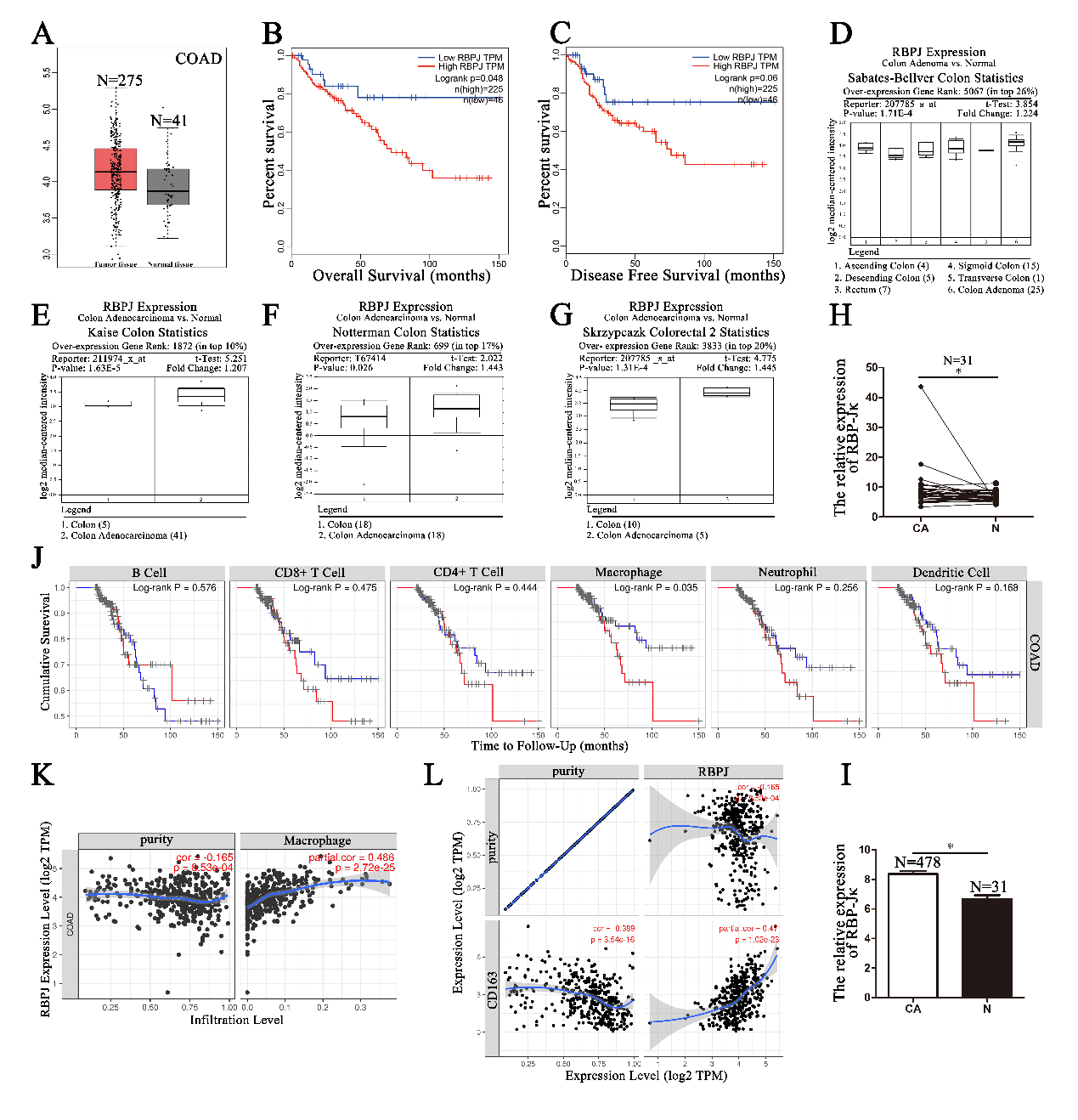
**

**Supplementary Figure 1. RBP-Jκ is overexpressed in colon cancer tissues in TCGA.** (A-C) TCGA data were analyzed with GEPIA. RBP-Jκ was overexpressed in colon cancer tissues versus normal tissues. High RBP-Jκ expression was associated with poor OS and DFS of patients. (D-G) RBP-Jκ overexpression in colon cancer tissues versus normal tissues (analyzed with Oncomine). (H) RBP-Jκ was overexpressed in colon cancer tissues versus paired paratumor tissues (analyzed with TCGA data). (I) RBP-Jκ was overexpressed in colon cancer tissues versus unpaired paratumor tissues (analyzed with TCGA data). (J) High macrophage infiltration was associated with poor survival (analyzed with TIMER). (K) RBP-Jκ expression was positively associated with macrophage infiltration (analyzed with TIMER). (L) RBP-Jκ expression was positively associated with CD163 (the marker molecular of type 2 macrophages; analyzed with TIMER). * *P*<0.05.

**
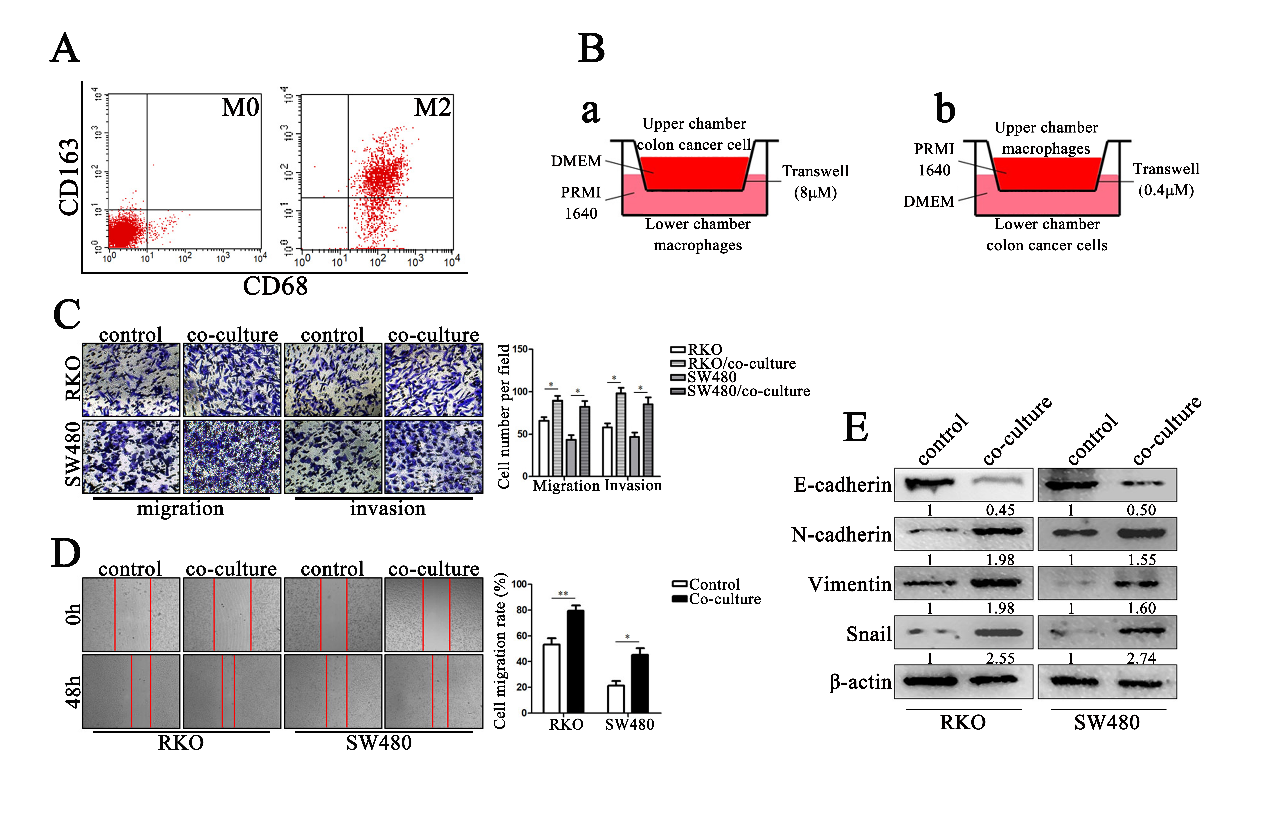
**

**Supplementary Figure 2. TAMs promote colon cancer cell metastasis.** (A) Flow cytometry. IL-4 and IL-13 were able to polarize macrophages into M2 type TAMs. (B) Transwell coculture system for colon cancer cell migration/invasion (a) and wound healing assay (b). (C) Transwell migration and invasion assays. TAMs prompted RKO cell and SW480 cell migration and invasion. (D) Wound healing assay. TAMs increased the migration rate of RKO cells and SW480 cells. (E) Western blot analysis. TAMs prompted RKO and SW480 cell EMT. * *P*<0.05 and ** *P*<0.01.

**
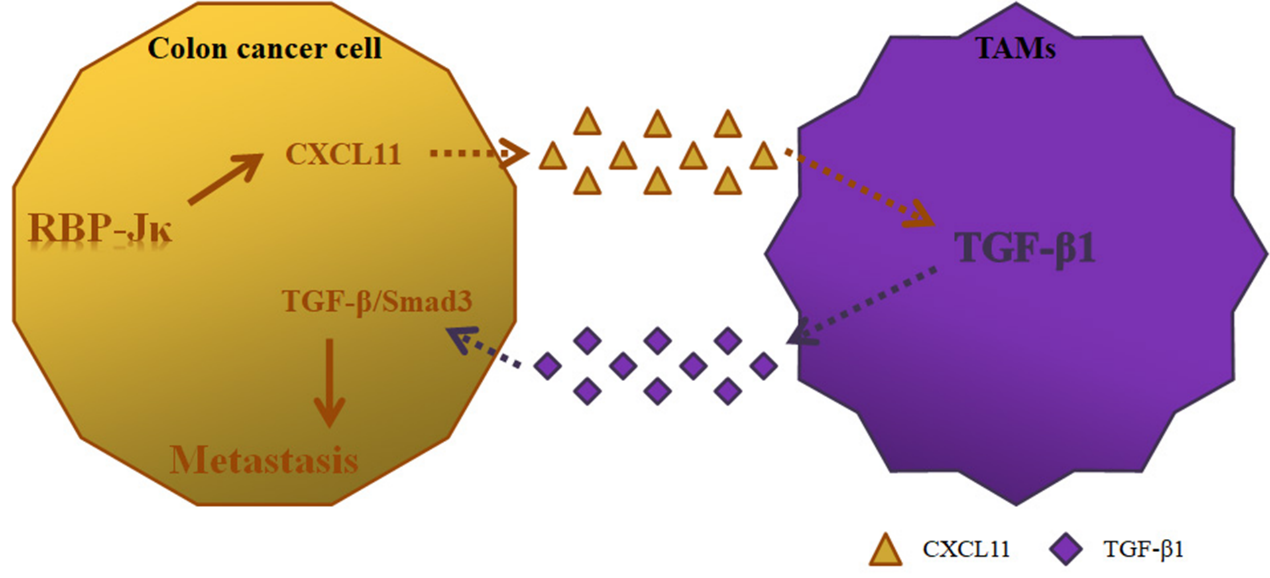
**

**Supplementary Figure 3. Schematic diagram summarizing the interactions between colon cancer cells and TAMs via RBP-Jκ, CXCL11 and TGF-β1**

**
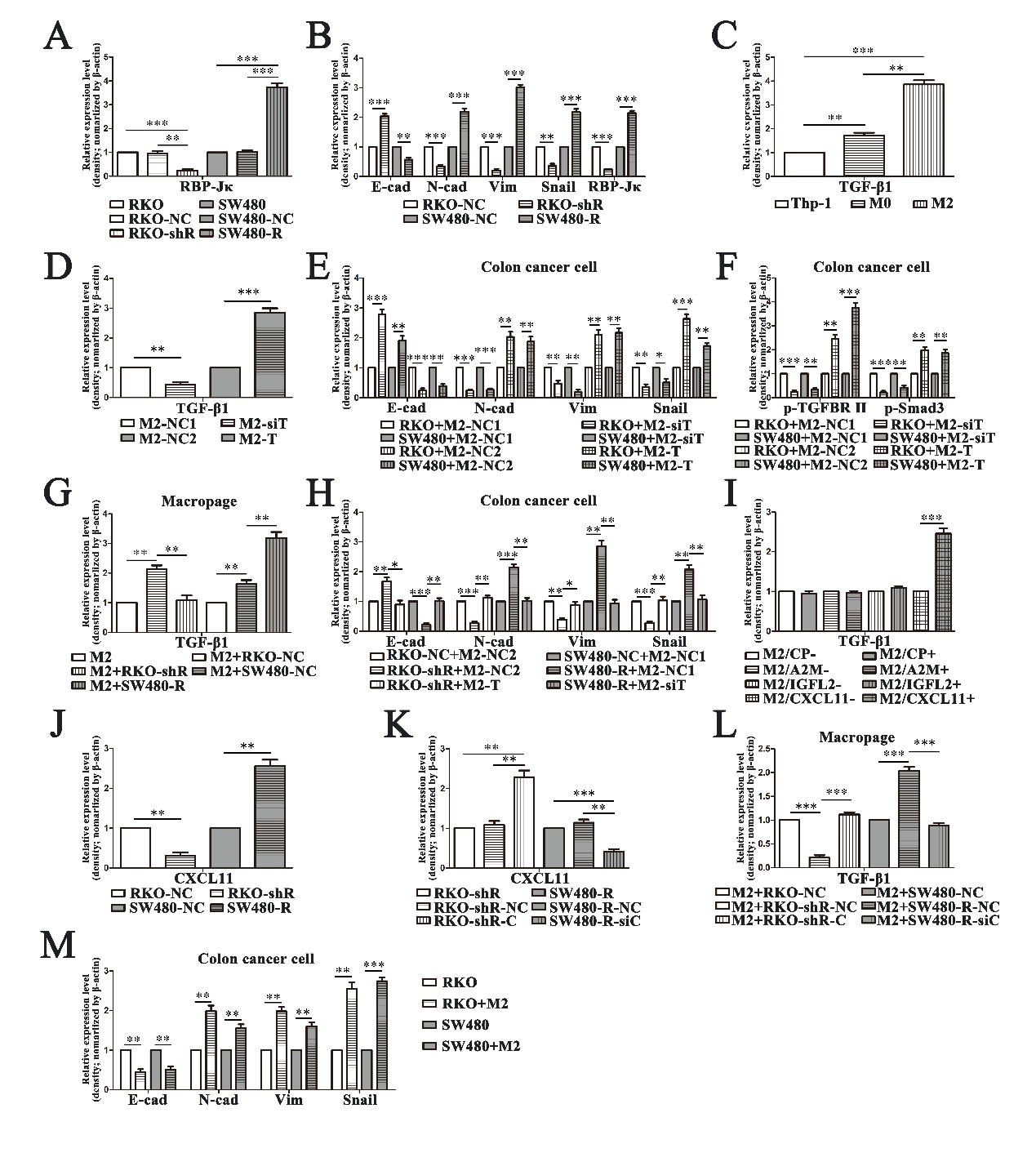
**

**Supplementary Figure 4. Histograms of Western blot quantitative analysis.** (A) Histogram of Figure 2A. (B) Histogram of Figure 2G. (C) Histogram of Figure 4A. (D) Histogram of Figure 4B. (E) Histogram of Figure 4F. (F) Histogram of Figure 4G. (G) Histogram of Figure 6A. (H) Histogram of Figure 6D. (I) Histogram of Figure 8D. (J) Histogram of Figure 8F. (K) Histogram of Figure 8I. (L) Histogram of Figure 8K. (M) Histogram of Figure S2E.
