# Supplementary figures and images for: Colon cancer cells secreted CXCL11 via RBP‐Jκ to facilitated tumour‐associated macrophage‐induced cancer metastasis
Source: J Cell Mol Med. 2021 Oct 16;25(22):10575–90. doi: 10.1111/jcmm.16989 (PMC8581314; doi:10.1111/jcmm.16989)

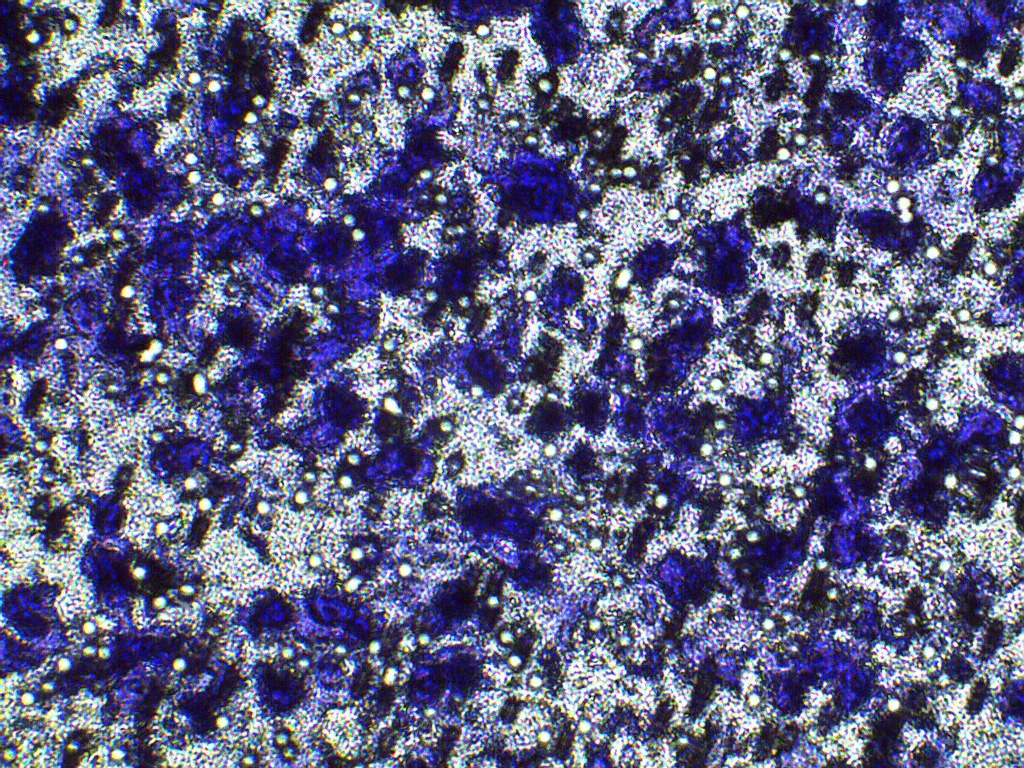

Supplement: Supplementary file 2 — Figure 2C [file JCMM-25-10575-s002.jpg]
